# Technical Design of a Low-Latitude Satellite Constellation for Ocean Observation with a Focus on Hainan Province, China

**DOI:** 10.3390/s25061710

**Published:** 2025-03-10

**Authors:** Lei Wang, Tianliang Yang, Tianyue Wang, Chengyi Wang, Ningyang Li, Xiao-Ming Li

**Affiliations:** 1 Key Laboratory of Earth Observation of Hainan Province, Hainan Aerospace Information Research Institute, Sanya 572029, China; wangchengyi@aircas.ac.cn (C.W.); ningyang799@gmail.com (N.L.); lixm@radi.ac.cn (X.-M.L.); 2 Aerospace Information Research Institute, Chinese Academy of Sciences, Beijing 100094, China; 3 Hainan International Commercial Space Launch Co., Ltd., Wenchang 571399, China; yangtlhainan@163.com; 4 Beijing Institute of Control Engineering, Beijing 100190, China; wwwwangty123@163.com

**Keywords:** low-latitude areas, Hainan Province, remote sensing satellite, constellation, ocean observations

## Abstract

Acquiring high-quality images from space at low-latitude areas is challenging due to the orbital requirements of the satellites and the frequent cloud coverage. To address this issue, a low-latitude remote sensing satellite constellation—the Hainan Satellite Constellation (HSC)—was conceived with a spatial coverage-priority concept. This constellation integrates sensors with multispectral, hyperspectral, radar, and Automatic Identification System (AIS) capabilities for marine vessels with an onboard image processing technology. The design is tailored to the tropical/subtropical region. Once HSC becomes fully operational, it will provide high-frequency coverage in low-latitude regions, with a primary focus on ocean observations. The first four optical satellites (HN-1 01/02 and WC-1 01/02) were successfully launched in February 2022. They boast unique application characteristics, including satellite networking for ocean observations over large areas, onboard image processing and modeling for ship detection, as well as the synergy of onboard sensors with optical and ship AIS capabilities. This study focuses on the technical design and proposes implementation strategies for HSC, encompassing its technical characteristics, composition, and capacity. Additionally, it explores the construction of this satellite constellation and its uses while providing insights into potential follow-up satellites.

## 1. Introduction

In recent decades, numerous satellite constellations have been launched with a focus on meteorology, oceans, and Earth applications. These include Synthetic Aperture Radar (SAR) constellations like RADARSAT and ICEYE, optical constellations, such as Rapid Eye, Planet Scope, and Digital Globe, and hybrid sensor constellations like COSMO SkyMed and Sentinels [1,2,3]. While most of these constellations were conceived for global-scale observations [4,5,6,7], a limited number were specifically designed for regional coverage [8,9].

To mitigate the deficiency in regional coverage of the above-mentioned constellations, some low-latitude satellites were designed, including Megha-Tropiques, CYGNSS, TROPICS, TRMM, LAPAN-A2, Jason-1, Jason-3, COSMIC-2, and FengYun-3G. Unlike the satellites with the global coverage, low-latitude satellites can observe the low-latitude regions efficiently, which is suitable for the applications related to atmosphere, ocean, soil, forest, etc., in these regions. For example, Megha-Tropiques [10,11], CYGNSS [12,13,14], TROPICS [15], TRMM [16,17,18], and LAPAN-A2 [19,20] were designed to handle rain prediction, soil moisture estimation, tropical rainfall measuring, and natural disaster monitoring in tropical countries. Jason-1 and Jason-3 were deployed with high-precision radar altimeters and a microwave radiometer, which is useful for measuring the sea level and modeling the sea surface [21,22,23]. The COSMIC-2 constellation integrates the radio occultation and space weather payload to observe the atmosphere and ionosphere and monitor space weather, such as tropical cyclones [24,25,26]. FengYun-3G, which was equipped with dual-frequency radar, a microwave imaging sensor, and a mid-resolution spectral imaging sensor, can recognize the change of weather in good time and precisely, especially precipitation [27,28]. The payloads of these satellites mainly focus on the radar, microwave imaging sensor, multispectral imaging sensor, and wireless, which are good at capturing the weather conditions, such as rain, atmosphere, storms, and space weather. But the imaging characteristics and resolutions of these sensors make them inappropriate for the typical application scenarios in the Hainan region, which include ship detection, fishery management, vortex monitoring, crop recognition, etc. To handle these tasks effectively, sensors with higher spectral and spatial resolutions and efficient imaging are required. This means that different payloads, including optical, radar, hyperspectral, and AIS, should be considered to collect more complicated information.

Advancements in remote sensing platforms and sensor technologies were reported [29,30], and proposals for systems integrating remote sensing and ship Automatic Identification System (AIS) satellites for enhancing ship detection revisit times have been put forth [31]. Comprehensive accounts of China’s remote sensing satellite constellations have also been documented [32,33,34,35,36,37,38,39].

China has made significant strides in developing Earth observation satellites with a focus on ocean observations. Three series of satellites, targeting oceanic color, marine dynamic environments, and marine surveillance were successfully launched [40]. However, the low-latitude regions of China, particularly in Hainan Province, remain insufficiently covered by dedicated ocean-focused spaceborne instruments, requiring further development of such specialized systems.

Situated in southern China, Hainan Province is a typical tropical/subtropical region surrounded by the sea, boasting rich natural resources in agriculture, marine, and forestry. Monitoring dynamic changes in these resources is crucial. Spaceborne remote sensing is a crucial tool for accurate and comprehensive data acquisition over extensive oceanic areas. The ongoing construction of the Hainan Free Trade Port demands precise spatial information, which can be retrieved through remote sensing and elaborated via data-driven tools. However, the low-latitude location of Hainan, coupled with the region’s frequent cloudy/rainy conditions, complicates the acquisition of precise spatial information. This significantly decreases the effective acquisition ability of remote sensing satellites in low-latitude regions. Consequently, current spaceborne instruments fall short of providing adequate coverage in such areas.

Due to the prevalent cloudy and rainy conditions in Hainan Province, the applicability of optical satellites is limited in this region. SAR-equipped satellites, benefiting from an active microwave remote sensing technology, can retrieve radar images in nearly all weather conditions [41,42]. Existing on-orbit SAR satellites turned out to be incapable of meeting the actual needs of resource monitoring for Hainan Province due to their incomplete coverage. Furthermore, considering that hyperspectral satellites generally have multiple wave bands with a narrow band range but can provide detailed information, a hybrid constellation including multispectral, hyperspectral, and SAR sensors is therefore required to meet the spatial information needs of Hainan and its surrounding areas.

In response to these challenges, the proposed Hainan satellite constellation (HSC) comprising ten satellites, including six optical satellites (HN-1 01/02/03/04 and WC-1 01/02), two hyperspectral satellites (SY-1 01/02), and two SAR satellites (SS-1 01/02), has been designed. HSC aims to address the specific climate characteristics, geographic location, and regional socioeconomic development needs. It can not only offer high-quality spaceborne information but also overcome the incomplete coverage of existing satellites.

This study details the technical design and proposed implementation strategies for the HSC, encompassing its technical characteristics, composition, capacity, construction, and application features. Additionally, it provides insights into the potential development of follow-up satellites.

## 2. Constellation Design

### 2.1. Mission Aims and Technical Requirements

Currently, low-latitude regions suffer a significant inadequacy of observation coverage because the observation focus of most polar-orbiting remote sensing satellites is the medium and high latitudes. To overcome the challenges associated with spatial information acquisition in low-latitude regions, HSC aims to establish a comprehensive hybrid-sensor satellite system capable of providing high-frequency and extensive observations in low-latitude regions when fully operational. The key technical requirements of the constellation include high spatial coverage, high frequency, and an application-oriented approach. As the main design concept is to enhance observational coverage, a focus on high spatial coverage is essential. Given the primary focus on ocean observation and, particularly, medium-size ship identification, the requirements, such as small-inclination orbit, a design concept of coverage priority, a sensor combination of multispectral, hyperspectral, SAR and ship AIS, onboard image processing technology for ship detection, swaying capability, spatial resolution, and swath width, were carefully considered during the planning, design and construction of HSC.

### 2.2. Constellation Technical Characteristics

As a low-latitude satellite constellation with the design concept of coverage priority, HSC features multispectral, hyperspectral, and SAR sensors, as well as AIS for integrated ocean observations. Onboard image processing technology is integrated for ship detection. Details of these characteristics are provided below.

#### 2.2.1. A Low-Latitude Satellite Constellation

Currently, most remote sensing satellites primarily focus on the medium and high latitudes, where the economy is the most active. Large-inclination and polar-orbiting satellites are often used in these regions, which results in a significant inadequacy of observation coverage in low-latitude regions. HSC addresses this issue by adopting a 30° inclination orbit. Its observation range is mostly limited between the Tropic of Cancer and the Tropic of Capricorn, which ensures the high coverage of low-latitude regions. In addition, the composition of 10 satellites can significantly improve the revisiting frequency and enhance the spatial coverage of the regions of interest.

#### 2.2.2. Design Concept of Coverage Priority

Through the development of remote sensing satellites, spatial resolution has always been one of key objectives. However, with the spatial resolution being improved, the swath width decreases accordingly, which results in the coverage and revisiting ability being decreased at the same time. Unlike the spatial-resolution-first concepts commonly used, HSC prioritizes coverage. Design elements, such as orbit inclination, swaying capability, swath width, imaging time per track, data download speed, and ground station distribution, are tailored for this specific need.

#### 2.2.3. Synergy of Sensors with Multispectral, Hyperspectral, SAR and Ship AIS

Considering the applicability of multispectral satellites is limited due to the prevalent cloudy and rainy conditions in this region, SAR satellites can retrieve radar images in nearly all weather conditions, and hyperspectral satellites can provide more detailed information. In addition, the AIS can help to achieve the accurate and real-time detection and tracking of ship targets. Synergy among multispectral, hyperspectral, radar sensors, and ship AIS integrates multi-modal spatial information for the ocean observations of Hainan and its surrounding areas to meet the demand for spatial information.

#### 2.2.4. Onboard Image Processing Technology and Dual-Line Array Delay System for Ship Detection

The remote sensing of ocean areas faces a fundamental challenge posed by seawater background information, which not only occupies data transmission bandwidth but also adds noise to the collected data. The integration of onboard image processing technology of the HN-1 01 satellite addresses this issue by facilitating ship detection and information acquisition, leading to a reduction in downlink data. The inclusion of a dual-line array delay imaging system enhances moving target extraction, streamlining the search for key targets. Users can opt to transmit only sliced images containing identified ships, instead of whole-track images, thereby strengthening the efficiency of ship identification and information transmission to ground stations.

Simulation has substantiated that, with a 30° inclination and a 500 km orbit height, three networking satellites can achieve daily coverage of the entire Hainan Province, which increases to 2–3 times a day in the key areas. Deploying 10 networking satellites with a wide swath width enables daily, high-coverage observations of low-latitude regions. Given the frequently cloudy and rainy weather conditions, the payloads of these satellites should include multispectral, hyperspectral, and SAR sensors [43].

A medium spatial resolution of 5 m proves sufficient for identifying ships exceeding 20 m in size. In the panchromatic spectrum, 20 m is in a critical state. Ships over 60 m can be detected in the panchromatic spectrum in calm sea conditions and ships over 100 m can be effectively detected.

### 2.3. Constellation Composition

**Figure 1:**
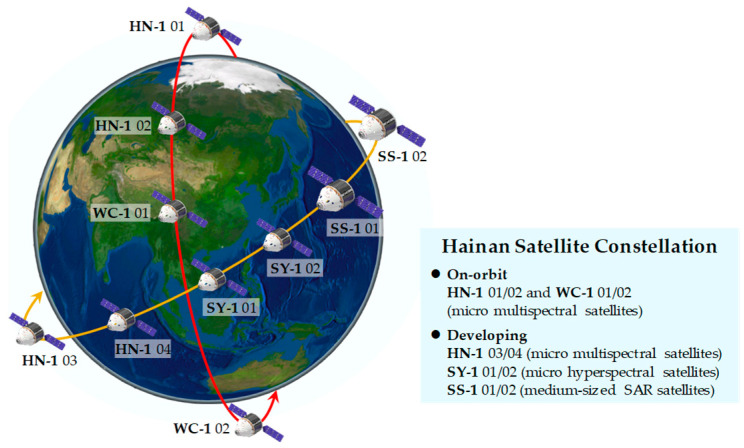
Architecture of the HSC, where the red line is the orbit of HN-1 01/02 and WC-1 01/02 at an inclination of 97° and the yellow line is the orbit of HN-1 03/04, SY-1 01/02 and SS-1 01/02 at an inclination of 30°.

The HSC design comprises ten satellites, including six optical satellites (HN-1 01/02/03/04 and WC-1 01/02), two hyperspectral satellites (SY-1 01/02), and two SAR satellites (SS-1 01/02). The project is implemented in two stages, the first one involving the development and launch of four optical satellites. However, due to commercial satellite project constraints, the lack of opportunities for launching small-inclination satellites resulted in the successful launch of four optical satellites (HN-1 01/02 and WC-1 01/02) into a Sun Synchronous Orbit (SSO) at an inclination of 97° and orbit height of 535 km in 2022. The remaining six satellites (HN-1 03/04, SY-1 01/02, and SS-1 01/02) will be developed in the second stage and will operate at a 30° inclination and an orbit height of ~500 km, as illustrated in Figure 1.

### 2.4. Constellation Capacity

**Figure 2:**
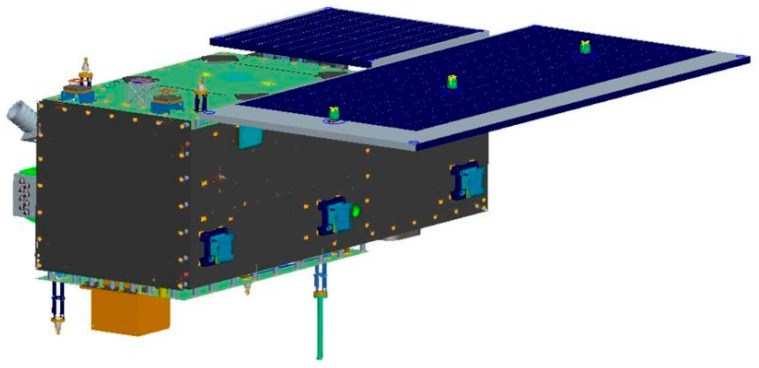
On-orbit status of the HN-1 01 satellite.

**Figure 3:**
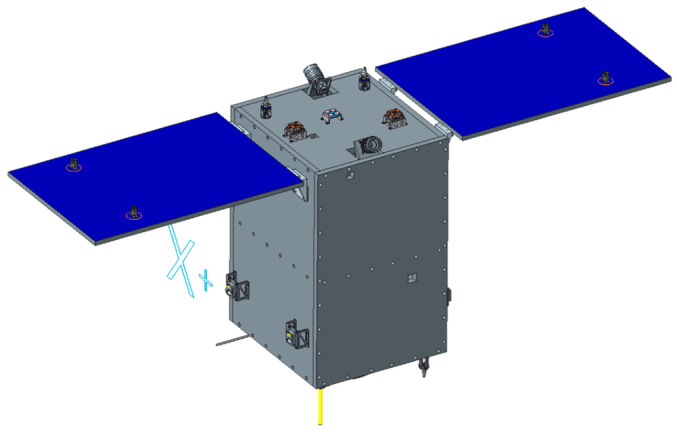
On-orbit status of the HN-1 02 satellite.

**Figure 4:**
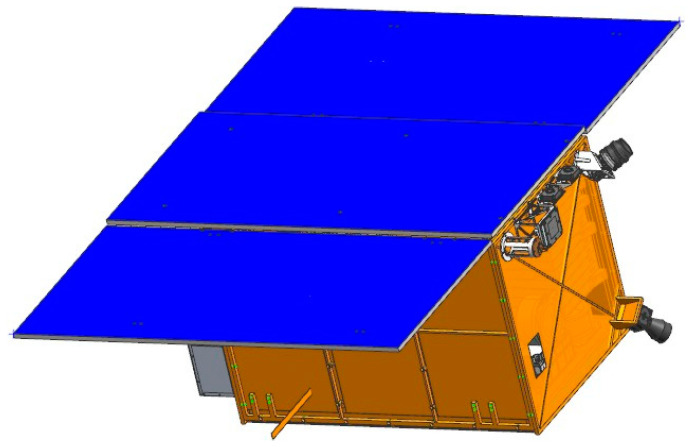
On-orbit status of the WC-1 01/02 satellite.

On 27 February 2022, the first phase of HSC, comprising the HN-1 01/02 and WC-1 01/02 satellites, was successfully launched at an inclination of 97° and an orbit height of 535 km. The on-orbit statuses of the HN-1 01/02 and WC-1 01/02 satellites are depicted in Figure 2, Figure 3 and Figure 4. Based on the phase differences in their orbits in late March 2022, using distinct swath widths, 110 km for HN-1 01, 115 km for WC-1 01/02, and 12 km for HN-1 02 and a 20° sway for each satellite, the satellite orbit simulations show that these four satellites will offer 3.5 days of observation coverage of Hainan Island and 5 days of observation coverage for regions adjacent to Hainan Island (100 km buffer).

## 3. Constellation Construction

### 3.1. Construction of the Satellite

The HN-1 01/02 and WC-1 01/02 satellites are integrated micro-satellites equipped with Earth observation and AIS information acquisition capabilities. They can be utilized for remote sensing and collecting ship information, such as speed, direction, and position.

In particular, HN-1 01 features a dual-line array wide camera with a spatial resolution of 5 m and a swath width of 110 km, enabling autonomous image processing for efficient ship detection and information transmission over large ocean areas. Moreover, HN-1 02 has a spatial resolution of 1.5 m and features both push-scan and video imaging working modes (12 km swath width for the push-scan working mode and 5.8 km × 3.3 km swath width for the video imaging working mode). The primary application of HN-1 02 is to monitor land and ocean properties as well as to retrieve ship information.

WC-1 01 and WC-1 02 are equipped with a panchromatic plus eight-band wide imaging camera, characterized by a spatial resolution of 5 m and a swath width of 115 km. Both satellites share identical technical specifications, making them suitable for networked observations.

The technical specifications of the HN-1 01/02 and WC-1 01/02 satellites are outlined in Table 1.

### 3.2. Payload

HN-1 01, specifically designed for ocean observation, is equipped with a dual-line wide array camera and AIS. It operates in various modes, including single-line array imaging, double-line array imaging, and double-line array detection. This versatility enables it to detect and retrieve ship targets. In single-line array imaging mode, the output is a remote sensing image acquired in low-power mode. In the double-line array imaging mode, the output is two remote sensing images. In double-line array detection mode, automatic ship detection is determined by image matching/comparison of two images with a temporal resolution of 5 s. Targets can be detected, and slice images of ship targets could be the main output.

HN-1 02 carries a main payload (a Bayer RGB push-sweep video integrated high-resolution optical camera) and a secondary payload (an AIS receiver). The high-resolution camera employs a Ritchey–Chretien optical system and a correction mirror, offering push-scan and video imaging working modes. The push-scan mode allows continuous imaging, while the video imaging mode captures 20 images per second with automatic exposure.

WC-1 01 and WC-1 02 feature a main payload (an area array CMOS optical camera) and a secondary payload (an AIS receiver). The CMOS optical camera supports panchromatic and multi-spectral push-sweep working modes, utilizing a refractive system and partition coating for imaging.

**Figure 5:**
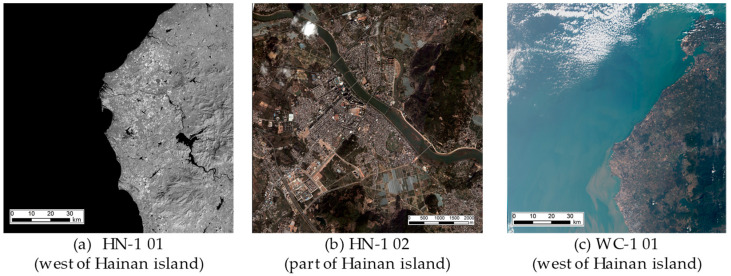
Examples of the observation data of HSC.

Partial images collected by these satellites are shown in Figure 5.

### 3.3. Construction of the Ground System

The ground system comprises four subsystems: satellite operation and control, data retrieval, data processing, and data management and distribution. Its primary function is to manage satellite operations, receive satellite data, process data, and distribute information. Satellite operational monitoring and control are conducted by the satellite mission ground monitoring and control center. This subsystem remotely controls satellites and receives telemetry data transmitted by the satellites. The data receiving subsystem, managed by remote sensing satellite ground stations in Beijing, Sanya, and Kashi, offers real-time data receiving services covering China and approximately 70% of the Asian landmass. The data processing subsystem processes raw data into Levels 0 and 1 after radiometric and geometric corrections. The data management and distribution subsystems handle metadata management, image data management, data service, and data portal nodes, based on four cloud servers for remote sensing data sharing services.

## 4. Application Characteristics

HSC focuses on Hainan Province, which is surrounded by the largest ocean area in China. Specific application scenarios determined the composition of HSC, sensor selection, and sensor technical specifications, including spectral band, spatial resolution, swath width, and swaying capacity. These parameters were designed to overcome the limited regional observation coverage, ensuring the constellation provides high-frequency and high-coverage observations of the province.

### 4.1. Satellite Networking for Observation of Large Ocean Areas

The four launched satellites possess unique characteristics and can work in synergy for improving the observational coverage of Hainan Province. HN-1 01 and WC-1 01/02, with their wide swath widths of 110–115 km, can be used for general surveys over large ocean areas to identify key research objects, such as ships. For instance, the size of a single-track image by HN-1 01 satellite is approximately 110 km × 1900 km, covering an area of ~200,000 km^2^. The multi-satellite network, i.e., by networking with WC-1 01 and WC-1 02, provides high-coverage observations of Hainan Province. Additionally, HN-1 02, with high spatial resolution and 4K HD video imaging capability, can be utilized to elucidate key objectives from a general survey. Integrating satellites into a network aid in achieving the mission’s key goals, including the discovery (via HN-1 01 and WC-1 01/02) and identification (via HN-1 02) of key targets.

### 4.2. Onboard Image Processing and the YOLO Model for Ship Detection

#### 4.2.1. Dual-Line Array Delay Imaging System

Through an analysis of the ship motion characteristics, it is found that the red and near-infrared spectral bands information has a higher contrast and is more useful for target detection. It is easy to detect ships and other moving targets with movement tracks in two different images by using information such as time differences among the two images. In addition, considering that ships are mainly affected by ocean waves and clouds in remote sensing images, the double linear array push-sweep mode is used to realize the observation effect of staggered time to the Earth, according to the location relationship of the detector (the scanning time interval of the double linear array is 5 s). Automatic detection of ships from the satellite images is completed by comparing two images acquired by the same camera at different times.

For HN-1 01, a dual-line array push-sweep imaging mode was specifically designed for onboard image processing for ship detection [44]. The advantages of the two-line array push-scan imaging model for HN-1 01 include: (1) onboard image processing for real time detection of an on-orbit moving ship by adopting the dual time array time-delay imaging scheme; (2) users have opts to transmit only sliced images containing identified ships, leading to a reduction in downlink data. This is useful for application requirements of real-time ship identity and sliced images with identified ships’ transmissions to ground stations.

The implementation process includes two steps. First, the near-infrared spectral bands are employed by sensors for ship detection. The uniform image background and lower reflectivity of seawater facilitate target separation. Second, the dual-line array push-sweep imaging mode is utilized for onboard ship detection. As ships dynamically move in the sea with constantly changing positions, image matching/comparison and motion detection algorithms can reduce the interference from waves and clouds and efficiently eliminate moving and stationary objects. This method lowers the false detection rate of ships and enhances the ship detection capacity. Specifically, with the dual-line array push-sweep imaging mode and the scanning time interval of 5 s, image matching/comparison and motion detection are applied to two consecutive images. Based on the ship geometric features database, the static background is removed and the false detection rate is reduced. During this process, the ship coordinates in the sliced images correspond to those in the original images. Results indicate the effectiveness of this method for onboard ship detection.

#### 4.2.2. Ship Detection with YOLOv5 Model

Except for the dual-line array push-sweep imaging mode of HN-1 01, the (You Only Look Once) YOLOv5 model [45] is employed for ship detection in the downloaded images of HSC satellites. It consists of three steps. First, the ship’s geometric features, including length, width, area, aspect ratio, and shape information from the remote sensing images, are recorded to construct the data set. Second, by integrating the basic geographic data and a pattern recognition method, the automatic segmentation of land, islands, and ocean is performed to reduce the negative impact of islands on ship detection. Third, the ship images are loaded into the system and the ship detection is performed. The output provides quantitative information on ship type, coordinates, geolocation (longitude and latitude), confidence, and size.

The data set used to optimize the YOLOv5 model contains the remote sensing images (HN-1 01/02 and WC-1 01/02) of various ships. The corresponding labels and positions of ships were marked manually to ensure the quality of samples. The proportions of training, validation, and test sets are 80%, 10%, and 10%, respectively. During the training procedure, the pre-train weights were first employed to initial the YOLOv5 model, which is helpful for promoting the convergence of the model. The mean square error and cross-entropy loss were both adopted to measure the difference between true labels and prediction. The stochastic gradient descent (SGD) [46] optimizer was adopted to update all parameters during back propagation.

**Figure 6:**
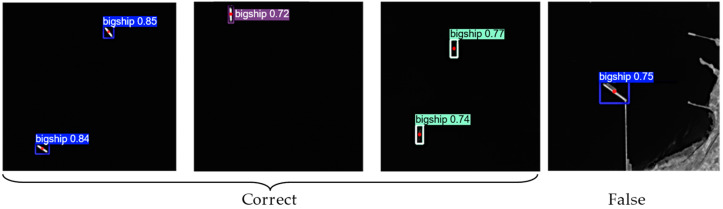
Visualization of detection results, where the types, confidences, and positions of ships were marked.

To evaluate the performance of the YOLOv5 model, the precision, recall, and f1-score were utilized. The confidences to determine the ship class and different ships were set to the optimized values of 0.65 and 0.20, respectively. After training, the best values of precision, recall, and f1-score reached 92.3%, 93.6%, and 92.9%, respectively. The visualization of several detection results is shown in Figure 6.

**Figure 7:**
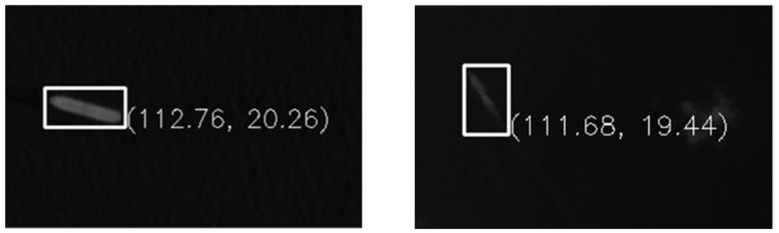
The smallest sizes of ships detected in the images with spatial resolutions of 5 m (**left**) and 1.5 m (**right**), where the rough longitudes and latitudes are marked on the right of targets.

In the real scenarios, the minimum size of the moving ships that could be detected correctly was 70 m (14 pixels) using 5 m spatial resolution images and 25 m (16.6 pixels) using 1.5 m spatial resolution images. Both examples are shown in Figure 7. However, the sizes of ships were roughly calculated according to the detection frame. Therefore, there may be some deviations in the results, which needs further evaluation.

### 4.3. Synergy of Onboard Sensors with Optical Camera and Ship AIS

The HSC combines an optical camera and ship AIS on a satellite platform for the detection, identification, and recording of dynamic ship information. All four launched satellites are equipped with remote sensing sensors and AIS onboard, enabling the retrieval of spatiotemporally collocated information regarding images and AIS. These technical capabilities are advantageous for obtaining ship parameters. After data processing, ships can be detected from images, and ship parameters, such as name, type, position, and speed, can be obtained by combining AIS (including commercial AIS information acquired from other AIS satellites) information with images when the ship position is matched. However, ships identified in the images without any retrieved AIS information require further post-processing analysis. As mentioned, HN-1 01/02 and WC-1 01/02 all have AIS onboard, which can collect approximately 800,000 items of AIS information every day in a 24 h regime.

### 4.4. Other Application Fields

The applications of the HSC can be broadened to other key fields, including ocean observations (e.g., maritime emergency searches and facilitating rescue operations, port development and monitoring, and marine logistics and transportation), natural disaster detection (e.g., oil spills, red tides, and storm surges), and coastal zone development monitoring (e.g., delta development and offshore wetland protection).

### 4.5. Application Cases

Through network observation of HN-1 01/02 and WC-1 01/02 satellites and utilizing the obtained remote sensing data and AIS information, application services have been launched in Hainan province, including monitoring of ships, analysis and prediction of target travel direction and monitoring of high-risk targets in the surrounding areas. It can meet the spatial data needs in key fields, such as the marine environment, maritime emergency search and rescue, and fishery information services.

Currently, the applications cases are mainly in Hainan province. But it is obvious that the applications of the HSC can be broadened to other low-latitude regions or even to global ocean monitoring, enhancing its applicability.

## 5. Follow-Up Development Plan

Due to the prevalent cloudy and rainy conditions in Hainan Province, the applicability of the launched satellites (HN-1 01/02 and WC-1 01/02) is limited in this region. The remaining six satellites of the HSC, including both hyperspectral (SY-1 01/02) and SAR (SS-1 01/02) satellites, will be developed during the second stage to achieve high-frequency coverage observations.

In particular, the two SY-1 hyperspectral satellites will be equipped with large-width and high-resolution hyperspectral cameras with push-scan imaging and staring video working modes. This imaging procedure is applicable for Earth observation purposes and can provide detailed spectral information for key applications in the fields of agriculture, marine and forestry resources.

The two SS-1 SAR satellites are planned to integrate Earth observations and ship AIS acquisition functions, in particular under adverse weather conditions. They will be equipped with C-band SAR with multiple imaging modes, such as sliding, bunching strip, and scanning. The two SAR satellites should help overcome the lack of remote sensing data caused by clouds and rain, while also facilitating the dynamic monitoring of ship, marine, and forestry resources.

## 6. Conclusions

In this article, HSC, a low-latitude satellite constellation, is designed to overcome the challenges in acquiring precise spatial information at low-latitude locations with frequent cloudy/rainy conditions. HSC prioritizes spatial coverage based on the small inclination and large swath width. It combines the multispectral, hyperspectral, radar, and ship AIS payloads and embeds the onboard real-time ship detection system. The constellation will provide the high-frequency coverage in low-latitude regions once it is fully operational.

The satellite networking of the launched HN-1 01/02 and WC-1 01/02 can enhance the observation coverage in Hainan Province, thereby facilitating ocean observation. In this constellation, the YOLOv5 algorithm for ship detection was integrated with the dual-line array push-sweep imaging mode. It realized both onboard image processing and the real-time detection of moving ships. Hence, only the sliced images containing identified ships can be transmitted to users, which reduces the downlink data effectively.

The YOLOv5 model is an efficient architecture, and the overall precision of it reached 92.3% after training with the ship images gathered in Hainan. In the real scenarios, the moving ships with the sizes of 70 m and 25 m can be recognized precisely in the images with the spatial resolutions of 5 m and 25 m, respectively.

The four launched satellites are all equipped with remote sensing sensors and AIS onboard, which enables the retrieval of spatial and temporal information regarding images and AIS. Synergy of onboard sensors with optical instruments and ship AIS can be effective for obtaining ship parameters when the ship position is matched.

However, given the limitations of optical remote sensing, SAR and hyperspectral satellites will likely to be added to HSC at a later stage. This will also help provide data-driven guidelines and support for the ongoing construction of the Hainan Free Trade Port.

## Figures and Tables

**Table 1:** Technical specifications of HN-01 01/02 and WC-01 01/02 satellites.

Satellite	Spatial Resolution (m)	Swath Width (km)	Spectral Range
HN-01 01	5	110	0.77–0.89 µm
HN-01 02	1.5	12 (push-scan) 6 × 3 (video)	0.4–0.7 µm
WC-01 01/02	5	115	0.45–0.90 µm

## Data Availability

Data are contained within the article.
